# Machine-Learning-Based Radiomics for Classifying Glioma Grade from Magnetic Resonance Images of the Brain

**DOI:** 10.3390/jpm13060920

**Published:** 2023-05-30

**Authors:** Anuj Kumar, Ashish Kumar Jha, Jai Prakash Agarwal, Manender Yadav, Suvarna Badhe, Ayushi Sahay, Sridhar Epari, Arpita Sahu, Kajari Bhattacharya, Abhishek Chatterjee, Balaji Ganeshan, Venkatesh Rangarajan, Aliasgar Moyiadi, Tejpal Gupta, Jayant S. Goda

**Affiliations:** 1 Department of Radiation Oncology, Tata Memorial Centre, Homi Bhaba National Institute, Mumbai 400012, India; 2 Department of Nuclear Medicine, Tata Memorial Centre, Homi Bhaba National Institute, Mumbai 400012, India; 3 Department of Pathology, Tata Memorial Centre, Homi Bhaba National Institute, Mumbai 400012, India; 4 Department of Radiodiagnosis, Tata Memorial Centre, Homi Bhaba National Institute, Mumbai 400012, India; 5 Institute of Nuclear Medicine, University College London Hospital, 235 Euston Road, London NW1 2BU, UK; 6 Department of Neurosurgery, Tata Memorial Centre, Homi Bhaba National Institute, Mumbai 400012, India

**Keywords:** glioma grade, radiomics, machine learning, classifiers

## Abstract

Grading of gliomas is a piece of critical information related to prognosis and survival. Classifying glioma grade by semantic radiological features is subjective, requires multiple MRI sequences, is quite complex and clinically demanding, and can very often result in erroneous radiological diagnosis. We used a radiomics approach with machine learning classifiers to determine the grade of gliomas. Eighty-three patients with histopathologically proven gliomas underwent MRI of the brain. Whenever available, immunohistochemistry was additionally used to augment the histopathological diagnosis. Segmentation was performed manually on the T2W MR sequence using the TexRad texture analysis softwareTM, Version 3.10. Forty-two radiomics features, which included first-order features and shape features, were derived and compared between high-grade and low-grade gliomas. Features were selected by recursive feature elimination using a random forest algorithm method. The classification performance of the models was measured using accuracy, precision, recall, f1 score, and area under the curve (AUC) of the receiver operating characteristic curve. A 10-fold cross-validation was adopted to separate the training and the test data. The selected features were used to build five classifier models: support vector machine, random forest, gradient boost, naive Bayes, and AdaBoost classifiers. The random forest model performed the best, achieving an AUC of 0.81, an accuracy of 0.83, f1 score of 0.88, a recall of 0.93, and a precision of 0.85 for the test cohort. The results suggest that machine-learning-based radiomics features extracted from multiparametric MRI images can provide a non-invasive method for predicting glioma grades preoperatively. In the present study, we extracted the radiomics features from a single cross-sectional image of the T2W MRI sequence and utilized these features to build a fairly robust model to classify low-grade gliomas from high-grade gliomas (grade 4 gliomas).

## 1. Introduction

Glioma is a broad term for primary brain tumors classified according to their presumed cell of origin [1]. The World Health Organization (WHO) has used histological grades to classify gliomas, ranging from grade I tumors with minimal proliferative capacity and a clinically benign course to aggressive grade IV tumors [2]. Despite accounting for less than 2% of all newly diagnosed cancers, gliomas are associated with significant morbidity and mortality [3,4]. Grade 4 gliomas, especially IDH wild grade 4 astrocytomas, are the most fatal, accounting for 70–75% of all gliomas with a median overall survival of 12 to 15 months and a 2-year overall survival of only about 30% [5,6].

Biologically gliomas are extremely heterogeneous tumors. The only way to discern the heterogeneity in gliomas is by studying the morphology of the resected tumor specimen, which sometimes may result in erroneous diagnosis even in the hands of expert pathologists. Imaging the brain is critical in brain tumor management because it allows for definitive diagnosis, tumor classification, management, and follow-up. MRI is the gold standard imaging approach for evaluating brain tumors [7]. Use of pre-operative MRI is a step toward using non-invasive techniques for characterizing and classifying gliomas. Additionally, different sequences of pre-operative MRI also assist the neurosurgeon in planning surgical resections. T1-weighted (T1W), T2-weighted (T2W), fluid-attenuated inversion recovery (FLAIR), T2W gradient echo, and post-contrast T1W images are the typically used MRI sequences [8]. Several adjunct imaging techniques, including diffusion-weighted imaging (DWI), perfusion-weighted imaging (PWI), and magnetic resonance spectroscopy (MRS), play an essential role in differentiating the various grades of tumors [9,10]. However, there still exists a gap in our knowledge regarding the utility of imaging to identify the highest-grade portions of gliomas on the local scale.

Heterogeneity is a well-known biological characteristic of tumor tissues. A significant tumor heterogeneity indicates unfavorable biology, an aggressive clinical course, and an unsatisfactory treatment response [11]. Deep learning and radiomics are two pillars of computational tumor image analysis [12]. Radiomics might be particularly useful for non-invasive glioma grading since it employs a voxel-by-voxel technique to turn sparse imaging data into big data (histogram, texture, and transformed features) [13,14].

Texture analysis, an emerging field of radiomics, is gaining popularity as a method for detecting heterogeneity from conventional tumor images obtained in day-to-day clinical practice [15]. Texture characteristics inside the tumor objectively capture intratumoral heterogeneity utilizing very advanced software techniques. It has shown potential in predicting pathologic features, response to therapy, and prognosis in various tumor types, including colorectal, head, neck, esophageal, lung, and renal cell carcinoma [16,17,18].

Differentiating between low- grade glioma (grade 2/3) and high-grade gliomas (grade 4) in brain tumors is critical for treatment and prognosis, since the degree of aggressiveness and infiltrative characteristics considerably impact the therapeutic strategy and prognosis. The spectrum of glioma grades is widespread with no clear distinction between low-grade gliomas vis à vis high-grade gliomas. Contemporarily, differentiating low-grade gliomas from high-grade gliomas has been based on the semantic radiological features that are discerned by the radiologist. This, however, may have some degree of subjectivity, as it depends on the experience of the radiologist and is inter-observer dependent. The wide spectrum of glioma grades and the subjectivity in differentiating these grades using preoperative MR-based radiologic features may have an overall impact on optimizing therapeutic decisions and prognosticating these tumors in day-to-day clinical practice. Using machine learning classifiers to model the radiomics features extracted from radiological images (MRI) is independent of inter-observer variation and can objectively classify low-grade gliomas (LGG—grades 2/3 gliomas) from high-grade gliomas (HGG—grade 4 astrocytoma). In radiomics-based glioma classification, machine learning methods such as support vector machines, random forest classifiers, etc., apart from deep neural networks are frequently employed to distinguish high-grade gliomas from low-grade ones. It is essential to emphasize, however, that this is an area of ongoing study, and further studies are required to prove the therapeutic value of this approach.

In this study, we applied robust machine learning classifiers to model the extracted radiomics features from a single sequence of magnetic resonance imaging (T2WMRI) of glioma patients to study whether the grade of glioma can be determined non-invasively. Our goal was to classify the WHO pathologic grade of the gliomas based on the radiomics features obtained from the imaging data. We sought to explore the accuracy of tumor radiomics using various machine learning classifiers in predicting the glioma grades on a T2-weighted MRI to demonstrate the effectiveness of these features using established performance metrics on the classification of each glioma’s histopathological grade and to use this approach as a robust imaging biomarker to guide therapeutic decisions as a prelude to possible clinical implementation in the future. The novelty of our approach was exemplified by extracting radiomics features from a single cross-sectional image of the T2W MRI sequence. These features were used to develop a robust machine learning model to differentiate LGG from HGG. This approach, once validated in a larger cohort, will have implications in day-to-day clinical practice in high-throughput tertiary care cancer centers. Additionally, we used five ML classifiers and compared their effectiveness using various diagnostic metrics to classify the grade of gliomas.

The novelty of the present study is that we extracted the radiomics features from a single cross-sectional image of the T2W MRI sequence and utilized these features to build a fairly robust model to classify low-grade gliomas from high-grade gliomas (Grade 4 gliomas) using five different machine learning classifiers in the test cohort. Secondly, isocitrate dehydrogenase (IDH) mutation analysis by immunohistochemistry was used in 64% patients for reclassifying the gliomas into low-grade and grade 4 astrocytomas.

## 2. Review of Literature

Gliomas are tumors of glial origin affecting the brain and spinal cord. According to World Health Organization (WHO) classification, gliomas are categorized into low- and high-grade based on the histopathology [2]. However, the wide spectrum of gliomas makes it difficult for even a pathologist to classify them correctly, often relying on molecular assays. Despite evolving imaging technology, non-invasive accurate prediction of glioma grade, survival, molecular status, and treatment response remains difficult. Biopsies continue to be the gold standard for histologic and genetic categorization, but they are invasive and expensive [19]. Machine learning (ML) and its offshoot, deep learning (DL), are prominent areas of artificial intelligence (AI). Rapid advancements in computing and imaging have significantly increased the potential for AI to influence neuroradiology diagnostics [20].

The development of radiomics, which extracts data from pictures by translating them into features measuring tumor characteristics, has accelerated the use of ML approaches to imaging, including radiomics-based analysis of brain tumors [21] In a study by Cho et al., five radiomics feature characteristics for glioma grading and three classifiers demonstrated an average AUC of 0.94 for training groups and 0.90 for test groups [22]. In several studies, the ML-based approach predicted glioma grades and expression levels of multiple pathologic biomarkers with good accuracy and stability [23]. Classifiers based on radiomics features have also shown benefits for predicting low-grades [24]. Although ML glioma grade prediction systems are becoming more widespread, they have yet to be implemented in routine clinical practice. With ongoing progress in computational processing, MR imaging texture analysis might become a powerful clinical tool for routine oncologic imaging.

Historically, the majority of radiomics-based ML and DL studies utilized image datasets from open sources collected across multiple institutions with heterogenous protocols and image quality. However, algorithms have been developed to reduce the heterogeneity to a certain extent in these datasets [20,22,25]. Radiomics models developed on these datasets may perform well in training and testing. Nevertheless, the results may not be reproducible in the real-world clinical practice, where images, imaging protocols and tumor presentations are heterogeneous. Therefore, the present study utilized image sets of a particular sequence (T2W) from a single institute to train the dataset.

## 3. Materials and Methods

Following approval from the Institutional Ethics Committee, 83 patients with histologically proven gliomas were included in this retrospective study. All patients underwent MRI to delineate the disease extent and treatment planning. T2-weighted MRI sequences were utilized for radiomics analysis. The MRI DICOM scans (Digital Imaging and Communications in Medicine) were obtained and pushed to the planning workstation, where the commercially available Texture Analysis research software TexRAD^®,^ Version 3.10 (Feedback Medical Ltd., Cambridge, UK, www.fbkmed.com) was used for tumor delineation and radiomics feature extraction. The glioma dataset had a total of 83 data points (patients) in two groups, namely low-grade gliomas (LGG or grades 2/3 gliomas) and high-grade gliomas (HGG or grade 4 astrocytoma), with 27 and 56 patients, respectively.

### 3.1. Inclusion and Exclusion criteria

#### 3.1.1. Inclusion Criteria

Age > 18 yrs;

Patients with histopathological diagnosis of glioma (wherever possible, immunohistochemistry of IDH was performed to augment the diagnosis);

Pre-operative MR images of the brain in DICOM format.

#### 3.1.2. Exclusion Criteria

Patients who did not have a histopathological diagnosis;

Patients whose preoperative DICOM MR images were not retrievable;

Patients who had multifocal/multicentric tumors in the brain on imaging.

### 3.2. The Overall Radiomic Workflow of the Study

**Figure 1:**
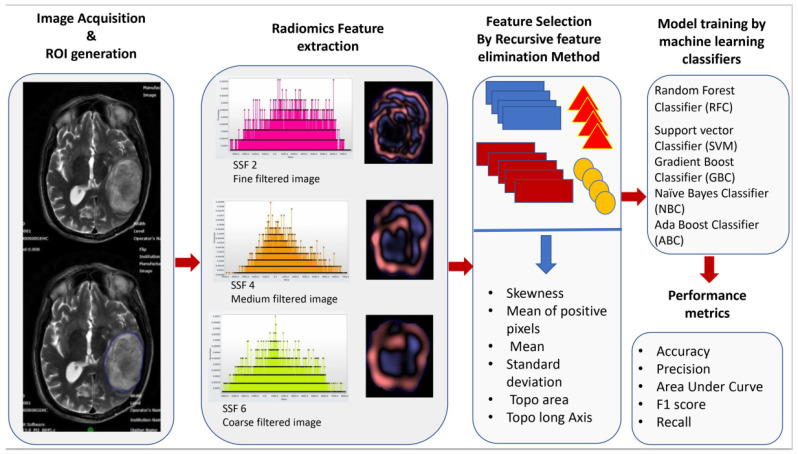
Workflow of image acquisition, segmentation, radiomics feature extraction, feature reduction, and building of the model by machine learning classifiers.

Figure 1 The radiomics pipeline represents the overall workflow of the study.

### 3.3. Magnetic Resonance Image Acquisition Protocol

Image acquisition protocol: Magnetic resonance imaging sequences of 83 patients were obtained at our institution using Ingenia 1.5T MRI—Philips™, Amsterdam, The Netherlands and GE Signa 3T M.R.I—General Electric™, Boston, MA, USA with a pre-fixed standard scanning protocol for brain tumor imaging. Patients were duly counseled before imaging. A 16-channel head coil was used. Axial T1 contrast (T1C) and T2W images were obtained from the vertex to the skull base, encompassing the whole brain. For contrast-enhanced T1W images, the rate of contrast injected was 3–4 mL/second and the total contrast injected was 0.1 mL/mmol/kg of bodyweight. These sequences were archived in the institutional Picture Archival and Communication System (PACS) and were exported to the radiomics (texture) analysis software (TexRAD™). The radiological features on the T2W MR images were evaluated and discerned by an experienced neuro-radiologist, TexRAD software was used for segmentation, and the texture features were extracted on the TexRad™ console. The acquisition protocol on the two MRI machines is described in Table 1.

### 3.4. MR Image Pre-Processing, Segmentation (ROI Generation)

Magnetic resonance imaging of the brain was acquired on two different MRI machines (1.5 Tesla Phillips™ and 3 Tesla General Electric™). The acquisition details of the MR images for the brain imaging protocol for both machines are explicitly described in Table 1. The resultant imaging protocol results in some imaging heterogeneity. Therefore, before segmentation and ROI delineation, image pre-processing was performed using the Laplacian of Gaussian (LOG) bandpass filters to remove the background noise (Gaussian filter) and to enhance the tumor edges (Laplacian filter). This allowed for the extraction of specific structures corresponding to the filter width. Spatial scale filters (SSF) used filtration values of 0, 2, 3, 4, 5, and 6 mm in width (radius), representing the increasingly coarser level of texture scales for first-order statistics. The use of a filtration algorithm before radiomics feature extraction helps in nullifying some of the effects of heterogeneous acquisition protocols and improves the robustness of the feature selection by removing the features affected by MR noise and imaging heterogeneity. Tumor segmentation and region of interest (ROI) delineation were performed using the semiautomated segmentation tool function of the software. Each ROI was drawn on the slice through the largest diameter of the target lesion around the peripheral margin. Air, streak artifacts, and dense calcifications were excluded from the ROI. However, tumor hemorrhage and necrosis if present were included within the ROI. The ROI contours and segmentation were separately verified by a neuro-oncologist with 10 years of experience and a neuroradiologist with 10 years of experience. The segmentation was verified by them individually, and any discrepancy was resolved by a consensus. For analysis, the final contours, as verified by the neuroradiologist, were considered.

### 3.5. MR Radiomics Feature Extraction (First-Order Texture Features and Shape Features)

**Figure 2:**
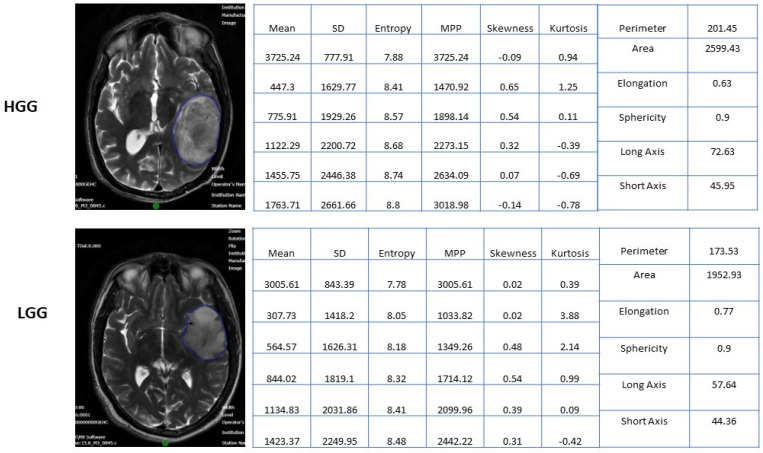
Texture and Shape features data of a representative patient samples patient of LGG & HGG).

The radiomics features were extracted from the segmented images using proprietary texture analysis research software (TexRAD™ Research Version 3.10, TexRAD Ltd., Cambridge, UK). The variables consisted of first-order texture features and shape (topographic) features. The first-order texture features are based on average pixel value. Intensity histogram analysis. They relate to gray-level frequency distribution within the region of interest, obtained from the histogram of pixel intensities. It is dependent on a single pixel value within the ROI rather than its interaction with neighboring pixels. The formulae for the extracted texture features are represented in Table 2. Shape features are extracted by three-dimensional surface rendering. These features include the descriptors of the three-dimensional size and shape of the ROI. Shape features are independent of the gray-level intensity distribution in the ROI. The shape features are calculated from the non-derived image (original image) and mask. The formulae for the extracted shape features are represented in Table 2. In the present study, each data point has 42 variables (features) extracted from the segmented images of the T2-weighted MRI sequences. Thirty-six first-order features were extracted using various spatially scaled filters (0, 2, 3, 4, and 6), and six shape (topographic) features were extracted without applying filters. All radiomics features were compared between high-grade gliomas (grade 4 astrocytoma) and low-grade gliomas (LGGs or grades 2/3 gliomas). Figure 2 shows the texture and shape (topographic) feature data of representative patient samples of LGG and HGG)

### 3.6. Data Normalization

Data normalization is a necessary step, as it gives equal weight to each variable, ensuring that no single variable steers the model performance in a direction because they are big numbers. Here, the min–max normalization (rescaling) technique is used to normalize the entire dataset. The general formula of min–max Z normalization for the range (0, 1) is given as x = (x − min(x))/(max(x) − min(x)). Here, x is the original value, and x is the normalized value.

### 3.7. Feature Reduction (Selection): Recursive Feature Elimination Method

Feature reduction or selection is a process used to minimize the number of parameters in a dataset while preserving as much variance as possible in the original dataset. This is performed before training the machine learning model, as it is a crucial step and avoids data overfitting, eliminates background noise by removing unnecessary attributes, identifies the most critical attributes responsible for the endpoint, reduces training time and computational resources, and improves the model’s overall performance.

Many feature classification algorithms have been developed for dimensionality reduction based on a wrapper and filtration approach as a precursor to model development. These algorithms include the least absolute shrinkage and selection operator (LASSO), leave-one-out cross-validation (LOOCV), the correlation-based feature selection algorithm, and the Recursive Feature Elimination (RFE) method. RFE is the most popular feature selection method, as it is easy to configure, flexible to use, and effective at selecting those features in a training dataset that are most relevant in predicting the target variable (endpoint). Moreover, this algorithm is a wrapper-type algorithm that can wrap around any ML model to produce the best feature set that gives the highest performance. By using the RFE method, features are initially ranked, and candidate subsets are generated. It removes the weakest feature (or features) until the specified number of features is reached, and then it refits the model. It determines which of the significant univariate variables were independent predictors of the endpoint of interest (i.e., class difference). Through this process, a list of accuracy values corresponding to each subset is produced. It is possible to establish a ranking of feature relevance that reflects their contribution to categorization.

Using the RFE method with classification algorithms, data dimensionality can be reduced significantly, resulting in increased processing efficiency. Hence, we used this method for radiomics feature selection in the present study.

In the present study, recursive feature removal with a random forest classifier was used (RF-RFE). By eliminating the least significant features, the RF model was trained and validated iteratively in the RF-FFE. After each cycle, the feature subset and model accuracy were kept. The feature subset with the best model accuracy was then chosen. Using the recursive feature elimination method, we selected six optimal features. The selected features included two topographic features (long axis and area) and four first-order texture features (skewness from an unfiltered image, MPP from a medium-filtered image, and mean and standard deviation from coarse-filtered images).

### 3.8. Training and Testing of Models

A 10-fold internal cross-validation was used on the training data. Here, the training data were split into ten subsets, one reserved for testing and the model being trained on the other nine subsets. This was then iterated ten times. These data were used to train five classification models: Random Forest Classifier (RFC), Support Vector Machine classifier (SVM), Gradient Boosting Classifier (GBC), Naive Bayes Classifier (NBC), and Ada-Boost Classifier (ABC). The models’ accuracy, sensitivity, and specificity were calculated from the confusion matrix. The area under the receiver operating curve (AUC) was also calculated, and the curve was plotted for all models. The most important features were identified using the random forest classifier, which is an ensemble machine learning algorithm of several individual decision trees that uses bootstrap aggregation or bagging. The data are sampled numerous times by RF, which then creates a unique prediction model for each sample. By combining the results of all the models, RF calculates the true mean value of the model. All of the models predict the individual outcome, and the ensemble outcome value of all the models is presented. Each tree gives a class prediction, and then, the class with the highest number of votes becomes the predicted result of the random forest model.

A support vector machine (SVM) builds a classifier to create a decision boundary between two classes of data known as a hyperplane. This hyperplane is orientated in the closest data points from each of the data classes, and these closest points are called support vectors. SVM is very useful, and it enables us to model higher-dimensional, non-linear models.

Gradient Boost classifiers are a family of machine learning algorithms that pool together several weak learning models to produce a robust predictive model. When performing gradient boosting, decision trees are typically used. The loss function or the difference between the actual and predicted classes is generally minimized by gradient-boosting classification methods using a logarithmic loss function.

The Naive Bayes classifier is a probabilistic graphical model for describing information about an uncertain domain where each node relates to a random variable and each edge reflects the conditional probability for the relevant random variables. The Bayes theorem is conditionally dependent on the structure of a collection of random variables selected in the model.

Ada-boost is an ensemble prediction algorithm. It combines various classifiers to improve the classifier accuracy. Ada-boost selects a training subset randomly. It iteratively trains the AdaBoost machine learning model by selecting the training set based on the accuracy of the prior training. It assigns a higher weightage to erroneously/incorrectly classified observations with a higher possibility of being classified in the subsequent iteration. Additionally, weight is applied to the trained classifier in each iteration based on how accurate it is. More weight will be given to the more accurate classifier. Until the maximum number of estimators is attained or the full training set accurately fits, the cycle is repeated.

### 3.9. Model Construction, Validation, and Performance of the Binary Classification Model

Model validation was performed by cross-validation. This is a method that examines the research model to achieve better residuals. We applied stratified ten-fold cross-validation and used a resampling method that uses different portions of the data to test and train a model on various iterations. Various prediction algorithms (Random Forest, Support Vector, Gradient Boosting, Naive Bayes, Ada-Boost) were used to predict responses using 10-fold validation, and they were studied compared with the ROC curve and AUC. Patient demographics were performed using SPSS v. 21.0. (IBM SPSS Statistics for Windows, v. 21.0, Armonk, NY, USA). Descriptive statistics were expressed as numbers and percentages for categorical variables and mean ± standard deviation or median (interquartile range) for continuous variables.

The prediction model assessment matrix was calculated using the following formulas:

Accuracy = ((TP + TN)/(TP + TN + FP + FN))(1)

Precision = (TP/(TP + FP)) = PPV(2)

Recall = (TP/(TP + FN))(3)

F1 = (2 × ((Precision × Recall)/(Precision + Recall))(4)

where, TP: true positives; FP: false positives; TN: true negatives; PPV: positive predictive value; FN: false negative; AUC: area under the curve; AUC of ROC; y-axis: true positive rate; x-axis: false positive rate

### 3.10. Baseline Demographics, Tumor, and Treatment Characteristics of the Study Cohort

The median age of the cohort was 50 years, with a higher male preponderance. The KPS was ≥80 in 75% of the patients. The most common site of the primary tumor was the frontal (39.8%), followed by temporal (34.9%), parietal (20.5%), and occipital regions (4.8%). Gross tumor resection was achieved in 39.8% of the patients, and the resection was subtotal in about 37.3%. The most common grade of the tumor was grade 4 (67.5%), followed by grades 2 (16.9%) and 3 (15.7%). After surgery, patients received adjuvant RT with a median dose of 59.4 Gy in 33 fractions. Table 3 shows the demographics of all patients in the study.

## 4. Results

### 4.1. Selected Features for Model Development

The top six features from the recursive feature elimination using a random forest algorithm were chosen as significant radiomics features for the endpoint of interest (i.e., class difference). The six most stable radiomics features were selected from the recursive feature elimination method and were used for model building using various machine learning tools with a 10-fold stratified cross-validation strategy. The selected features included two topographic features (long axis and area) and four first-order texture features (skewness from unfiltered image, MPP from medium filtered image, and mean and standard deviation from coarse filtered images). The top feature that was most valuable was the shape (topographic) feature’s long axis. The next most efficacious features were the first-order texture features characteristic of the intra-tumoral area.

### 4.2. Test Performance Measures Using Various Machine Learning Classifiers

**Figure 3:**
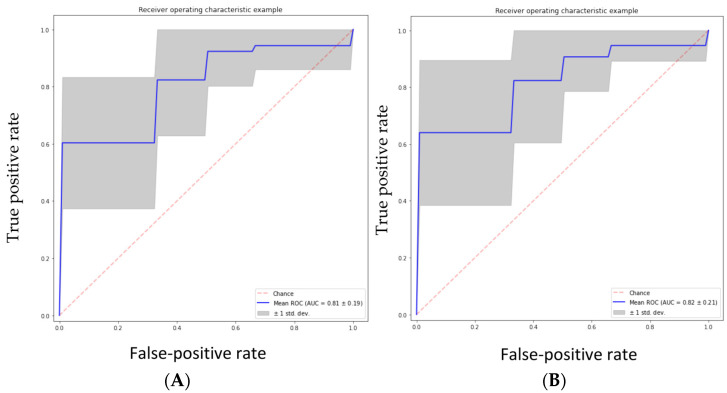

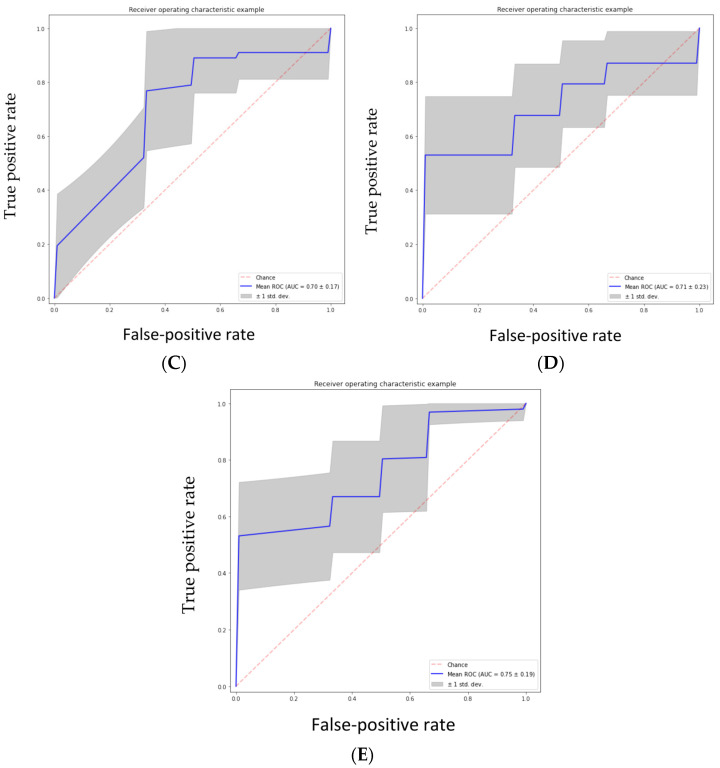
(**A**) Random forest classifier (RFC) model, (**B**) support vector classifier (SVC) model, (**C**) gradient boosting classifier (GBC) model, (**D**) naive Bayes classifier (NBC) model, (**E**) AdaBoost classifier (ABC) model.

**Figure 4:**
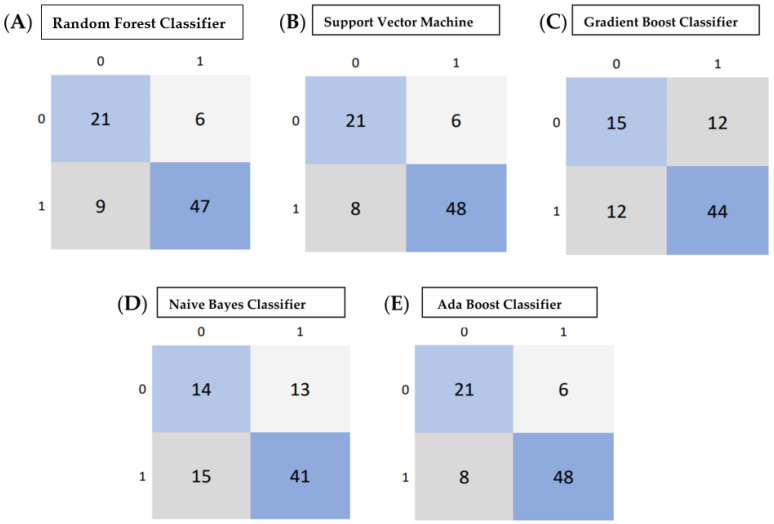
(**A**–**E**) Confusion matrix calculated from various machine learning algorithms for classifying low-grade gliomas (grades 2/3 gliomas) and HGG (grade 4 astrocytoma). (**A**) Confusion Matrix of Random Forest Classifier, (**B**) Support Vector Machine, (**C**) Gradient Boost Classifier, (**D**) Naive Bayes Classifier, (**E**) Adaboost Classifier. Within each matrix, the matrix row represents the instances in the actual ground true class, while each column represents the instances in the predicted class. The main diagonal shown in light blue represents the number of data points that were classified correctly.

The performance of the five classifiers using ten-fold cross-validation is shown in Table 4. Of the five classifier models, the random forest model was found to be the most stable and performed better than the other four classifier models for all the performance metrics in differentiating the grades of gliomas. The RF classifier achieved a predictive performance (AUC: 0.81, accuracy: 0.83, precision: 0.85, recall: 0.93, f1 score 0.88) for the test cohorts. The support vector machine classifier (SVM) also performed well with an AUC of 0.82, accuracy of 0.82, and precision of 0.85; however, the recall metric and f1 scores were slightly lower than the RF classifier (recall: 0.91 and f1 score: 0.87). The performance of the other three classifiers (GBC, NBC and ABC) was inferior, with an AUC and accuracy of <0.80. Table 4 shows the prediction model performance for differentiating low- and high-grade gliomas using different performance metrics. Figure 3A–E show the ROCs for the five classifiers in the test cohort. Figure 4A–E show the confusion matrixes for the five classifiers in the test cohort. Within each matrix, the horizontal row represents the actual ground true class, while each column represents the predicted class. The main diagonal shown in light blue represents the number of data points that were classified correctly.

The area under the curve (AUC) was calculated from various machine learning algorithms for classifying low-grade gliomas (grades 2/3) and high-grade gliomas (grade 4 astrocytoma).

## 5. Discussion

Brain tumors are frequently heterogeneous, with diverse histopathologic features making it challenging to estimate the grade of the tumors [26]. Gliomas may exhibit a range of traits, including both low- and high-grade characteristics. Currently, the reference standard for defining brain neoplasms is based on histopathologic examination following surgical biopsy or resection, although this has drawbacks such as sampling errors and interpretation uncertainty [27,28]. Radiomics has emerged as a powerful tool to quantify the characteristics of tumors in a non-invasive manner and can be utilized to differentiate between low- and high-grade gliomas. However, the radiomics approach may result in high-dimensional data that would be difficult to interpret.

A machine learning approach using radiomics features can be used to compute high-dimensional features from in vivo imaging modalities, which in turn were used to differentiate between high-grade glioma (GBM) and low-grade gliomas in this study. Our study used five classifiers to distinguish between low-grade and high-grade gliomas. Among all algorithms, the random forest classifier performed the best, with an AUC of 0.81, an accuracy of 0.83, a precision of 0.85, a recall of 0.93, and an f1 score of 0.88 for the test cohorts (Table 4). The results of our study have been corroborated by a group from South Korea who tested three different classifiers (RFC, SVM, and logistic regression) using 468 radiomics features extracted from multi-modal MRI imaging (T1-weighted, T1-contrast enhanced, T2-weighted, and FLAIR images). This study also showed the RFC classifier as the best and most stable performer compared to SVM and logistic regression in differentiating high-grade gliomas from low-grade gliomas. The averaged-out AUC for all three classifiers was 0.9030, while the AUC of RFC was 0.92, which was much higher than our study. The study by Cho et al. had a large cohort size of 285 patients (HGG: 210 and LGG: 75). On the contrary, the cohort size in our study was only 83 patients (HGG: 56 and LGG: 27).

Multiple other studies have attempted to differentiate HGG from LGG, albeit using different approaches. In a study by Zacharaki et al., 98 patients with brain tumors included metastases, meningiomas, and gliomas; the highest accuracy was noted for metastasis (91.7%) and low-grade gliomas (90.9%). The classification accuracy was less for GBM, where it was noted that 29.4% were classified as grade 3 and 29.4% as metastasis. This study achieved an accuracy of 0.878 and an AUC of 0.896 using SVM-based recursive feature elimination (SVM-RFE) with leave-one-out cross-validation (LOOCV). The leave-one-out cross-validation approach in the above study may have led to overfitting, resulting in erroneous interpretation. Studies have demonstrated that SVM-based categorization of texture patterns is a promising method for establishing objective and quantitative evaluations for brain tumors [29]. In contrast to Zacharaki’s study, which adopted an SVM-RFE with LOOCV approach for feature selection and an SVM classifier in discriminating HGGs from LGGs, we utilized recursive feature elimination using a random forest algorithm for feature reduction and five different machine learning classifiers with a stratified 10-fold cross-validation approach to reduce overfitting.

MRI sequences using relative cerebral blood volume (rCBV) measurements and metabolite ratios from proton MR spectroscopy have been used to distinguish between HGG and LGG with a sensitivity of 0.950 and a specificity of 0.575 [30]. Togao et al. utilized intravoxel incoherent motion (IVIM) MR imaging in 45 patients and attained a sensitivity of 0.96, specificity of 0.81, and AUC of 0.95 with conventional image-processing techniques [31].

We found six significant and stable radiomics features through ten-fold cross-validation. Two shape features (area and long axis) and four first-order texture features were selected and used to build the model for discriminating HGG from LGG performed consistently. Glioma shape is a well-known factor associated with malignancy, as irregular tumor shape is often associated with tumor aggressiveness and poor prognosis [32]. We found that the shape features of the tumor on T2W MR images were important in determining the glioma grades. Apart from shape features, tumor heterogeneity is an important predictor for prognosticating a disease, which can be quantified by texture features. In our study, we found that the four first-order texture features were important in classifying glioma grades. Other radiomics studies have shown features such as GLCM to correlate various clinical endpoints in various tumors [33,34,35,36,37].

Different techniques of machine learning applied to various imaging modalities have been studied for glioma grading. We applied five different types of ML classifiers (RFC, SVM, naive Bayes, AdaBoost, and gradient boost) to differentiate glioma grade as a binary classification problem (LGG vs. HGG). In our study cohort, we found that RFC and SVM classifiers performed the best for classifying LGG and HGG using various diagnostic metrics with an accuracy of 0.83 and 0.82 and AUC of 0.81 and 0.82, respectively (Table 4) On one end of the spectrum, simple statistical tools such as logistic regression have been used to classify gliomas with reasonable accuracy of 93%, a sensitivity of 97%, a negative predictive value of 99%, and an AUC of 0.94 [38], while on the other end of the spectrum, complex and sophisticated ML tools such as RFC and SVM have been used not only for predicting glioma grades but also for prognosticating the disease. Logistic regression is a too simplistic model and suffers from overfitting, especially in high-dimensional data, which is a norm in radiomics studies. On the contrary, ML tools such as SVM and RFC are more adept at handling high-dimensional data, which is usually the norm in radiomics studies. SVM classifiers have been used to classify gliomas on resting-state functional MRI images, although these MR sequences are seldom used in clinical practice [39]. SVM classifiers have been used with reasonable accuracy (AUC: 0.987) for diagnosing low-grade vs. high-grade and grade 3 vs. 4 gliomas [40]. A group from South Korea used multiple sequences of MRI and extracted radiomics features from the Brain Tumor Segmentation 2017 Challenge. LR, RFC, and SVM machine learning algorithms were used in the analysis to model radiomics features for classifying HGG from LGG in a cohort of 285 patients after splitting the data into training and test cohorts [22]. Five significant radiomics features were selected for the machine learning classifiers, and the three classifiers showed an average AUC of 0.94 for the training cohorts and 0.90 (logistic regression 0.90, support vector machine 0.88, and random forest 0.92) for the test cohorts in the above study. The findings of our study corroborated the results obtained by Cho et al. [22]. We observed that both RFC and SVM ML tools performed relatively well in differentiating LGG from HGG for the various diagnostic metrics (Table 4). In contrast to Cho’s study, we used five ML algorithms with 10-fold internal cross-validation to model the radiomics features obtained only from a single T2W MRI sequence. Using a single MR sequence reduces cost and time; moreover, it can be scalable for clinical implementation once we validate our observations in a larger cohort of patients, which is a work in progress.

Evolution in deep learning (DL) algorithms such as convoluted neural networks (CNN), has led to their use classifying diseases, predicting treatment response, and prognostication of disease [41]. DL approaches have shown promise in tumor grading and diagnostics and prognostication of disease [42,43,44,45]. Deep learning algorithms augment traditional neural networks by adding hidden layers to network architectures between input and output layers while modeling more complex and nonlinear situations. The advantage of the DL approach is that it does not require human intervention to specify a set of features a priori but can implicitly learn the features relevant to the given problem and thus can be effective for radiomics research. However, another side of the DL approach is that these algorithms are extensively data-hungry and require large datasets to train and validate compared to conventional machine learning classifiers, which require limited imaging datasets. Additionally, issues arise while fine-tuning hyperparameters. Although these issues are challenging to deal with, we feel that DL approaches would give more robust and meaningful results once trained and validated on large datasets. Our future endeavor is to develop an image bio-banking system that will feed a large number of image datasets for training the DL algorithm to classify LGG from HGG with a high degree of accuracy.

Our study has a few limitations. Being a retrospective study, patient image datasets for developing the radiomics model were obtained from 2014 to 2016, when molecular studies (IDH) were being introduced in our institute. Hence, we could obtain the IDH mutation status in only 64% (53/83) of patients. Therefore, the gliomas were reclassified based on histomorphology and IDH status, while in 36% of patients, gliomas were classified based only on histomorphological features. This was mainly because the tissue material was extensivelydegraded to perform any IDH studies. Secondly, we had only limited imaging datasets; therefore, we could not perform an external validation of the results. However, we tried to reduce overfitting of the data by an internal 10-fold stratified cross-validation technique. However, we presume that this may hinder the applicability of our approach to a new dataset. Additionally, there was a class imbalance between two classes (HGG: 56 and LGG: 27) that could skew the results. The relatively small sample size of our study also limited the use of deep learning algorithms, which are data-hungry and require a massive number of image datasets, which would not have been possible without the pooling of image data from multiple institutions, which in itself could have introduced a confounding factor of image heterogeneity, as different institutions have different imaging protocols, resulting in variability and generalization gaps in the predictive model. Regardless of the heterogeneity in MR acquisition parameters, we were able to achieve a relatively fair bit of accuracy in developing a reasonably robust model, suggesting that this would consequently have a clinical implication if validated in a large cohort of patients in real-world clinical practice. Additionally, the current methodology of using internal cross-validation has the limitation of inflating the performance metrics. However, with a limited sample size, we thought that the internal 10-fold cross-validation would be the best strategy for model development. One of the strengths of the study was that the reproducibility of the ROI was verified by an experienced neuroradiologist blinded to the results of the subgrouping. The model developed in the current study is planned to be tested on an independent validation cohort and subsequently on a more extensive imaging database.

## 6. Summary and Conclusions

Machine-learning-based radiomics approaches can provide a simple and non-invasive method for predicting glioma grades preoperatively on MRI, with favorable predictive accuracy and stability. ML tools are increasingly being used to not only predict the grade of glioma but also prognosticate gliomas. These tools are increasingly used in brain tumor research but require extensive validation studies for their incorporation in clinics for making therapeutic decisions. Apart from validation studies, some of the impediments to the clinical translation of ML are the low reporting quality in glioma grade prediction and lack of reproducibility. Currently, efforts are focusing on creating uniformity in reporting guidelines and radiomics quality scores, which would provide the opportunity for the implementation of radiomics-based ML algorithms for clinical implementation.

In the present study, we selected the six most stable radiomics features (four texture features and two topographic features as obtained from the recursive feature elimination (RFE) algorithm, and we used these features to build a robust model using five different machine learning classifiers to model the radiomics features for differentiating low-grade gliomas (grades 2/3) from grade 4 astrocytomas. Our study showed that both the random forest and support vector machine classifiers were the most accurate in predicting the grade of glioma, with an accuracy of 83% and 82% and an AUC of 0.88 and 0.87, respectively. Although both models had the same precision (85%) in classifying LGG from HGG, the performance of the random forest model was better than the support vector machine in terms of recall (93% vs. 91%). The performance of the other three classifiers, namely AdaBoost, gradient boost, and naive Bayes, was inferior (accuracy: 66–74% and 70–74%). We acknowledge that the results of the present study are based on a limited dataset. We plan to use a larger image dataset to reduce the bias of oversampling of a minor class to balance the sample ratio and to include an external validation set. The results of the study, once validated on an external dataset, will be used to scale up for clinical research.

## Figures and Tables

**Table 1:** T2W Magnetic resonance imaging acquisition parameters for gliomas.

MRI Machine	Sequence	FOV (cm)	Matrix	NEX	Slice Thickness (mm): Slice Gap (mm)	TR	TE
GE Signa 3T	Axial T2W	24	320 × 224	1	5:1.5	4080	90
Philips Ingenia 1.5T	Axial T2W	23 (AP) 18.5 (RL)	448 × 304	2	5:1	6000	107

**Table 2:** Radiomics features extracted from T2W MR images.

1st Order Texture Feature	Formulae	Remarks
Mean	$Mean=\frac{1}{N_{p}}\sum_{i=1}^{N_{p}}X\left(i\right)$	Mean represents the average gray-level intensity within the ROI.
Standard deviation	$SD=\sqrt{\frac{1}{N_{p}}\sum_{i=1}^{N_{p}}(X\left(i\right)-\bar{X}{)}^{2}}$	*SD* represents variation from mean gray-level value; *SD* is small if image is homogenous.
Skewness	$skewness=\frac{{µ}_{3}}{{\sigma^{3}}_{}}=\frac{\frac{1}{N_{p}}\sum_{i=1}^{N_{p}}(X\left(i\right)-\bar{X}{)}^{3}}{{\left(\sqrt{\frac{1}{N_{p}}\sum_{i=1}^{N_{p}}(X\left(i\right)-\bar{X}{)}^{2}}\right)}^{3}}$	Skewness is the symmetry of intensity values in an image (ROI). It is zero if the histogram is symmetrical.
Entropy	$Entropy=\sum_{i=1}^{N_{p}}p\left(i\right){log}_{2}\left(p\left(i\right)+\epsilon \right)$	Entropy represents irregularity or randomness of intensity value distribution in the ROI.Here, *ϵ* is an arbitrarily small positive number (≈2.2 × 10^−16^).
Kurtosis	$Kurtosis=\frac{{µ}_{4}}{{\sigma^{4}}_{}}=\frac{\frac{1}{N_{p}}\sum_{i=1}^{N_{p}}(X\left(i\right)-\bar{X}{)}^{4}}{{\left(\frac{1}{N_{p}}\sum_{i=1}^{N_{p}}(X\left(i\right)-\bar{X}{)}^{2}\right)}^{4}}$	Kurtosis is an indication of histogram flatness.
Shape Feature	Formulae	Remarks
Elongation	$Elongation=\sqrt{\frac{\lambda_{minor}}{\lambda_{major}}}$	Elongation shows the relationship between the two largest principal components in the ROI shape. *λ_major_* and *λ_minor_* are the lengths of the largest and second largest principal component axes. The values range between 1 (where the cross-section through the first and second largest principal moments is circle-like (non-elongated)) and 0 (where the object is a maximally elongated).
Area	$A_{pixel}=\sum_{k=1}^{N_{v}}A_{k}$	The surface area of the ROI A pixel is approximated by multiplying the number of pixels in the ROI by the surface area of a single pixel *A_k_*.
Sphericity	$Sphericity=\frac{2\sqrt{\pi A}}{P}$ where $A=\pi r^{2}$	*Sphericity* is the ratio of the perimeter of the tumor region to the perimeter of a circle with the same surface area as the tumor region and therefore a measure of the roundness of the shape of the tumor region relative to a circle.
Short axis	$Short\;axis=4\sqrt{\lambda_{minor}}$	This feature yields the second largest axis length of the ROI-enclosing ellipsoid and is calculated using the largest principal component *λ_minor_*.
Long Axis	$Long\;axis=4\sqrt{\lambda_{major}}$	This feature yields the largest axis length of the ROI-enclosing ellipsoid and is calculated using the largest principal component *λ_major_*.
Perimeter	$P_{i}=\sqrt{{{(a}_{i}-b_{i})}^{2}}$ where $P=\sum_{i=1}^{N_{f}}P_{i}$ *a_i_* and *b_i_* are vertices of the ith line in the perimeter mesh. Total perimeter is then obtained by taking the sum of all calculated sub-areas	

**Table 3:** Demographics and tumor and treatment characteristics (*n* = 83).

Patient Characteristics	Frequency (%)
**Age (in years)**	
Median (interquartile range)	50 (38–61)
**Sex**	
Male	51 (61.4%)
Female	32 (38.6%)
**Karnofsky performance status**	
≥80	62 (74.7%)
<80	21 (25.3%)
**Site**	
Frontal	33 (39.8%)
Parietal	17 (20.5%)
Temporal	29 (34.9%)
Occipital	4(4.8%)
**Surgery**	
Gross total resection	33 (39.8%)
Near total resection	16 (19.3%)
Subtotal resection	31 (37.3%)
Biopsy only	3 (3.6 %)
**Grade**	
Low-grade gliomas (glioma grades 2/3)	27 (32.6%)
High-grade glioma or grade 4 astrocytoma	56 (67.5%)
**IDH status**	
IDH + ve (mutant)	19
IDH − ve (Wildtype)	331
IDH Uninterpretable	30
IDH unavailable *	
**Median dose of radiotherapy**	59.4 Gy

* IDH: isocitrate dehydrogenase; IDH was not available, as the biopsy material was degraded; hence, IHC could not be performed on the tissue samples.

**Table 4:** Prediction model performance from selected radiomics features for classifying low-grade gliomas form GBM.

Algorithm/ Model	Validation	Class Probability		Accuracy		Performance Metrics		
		0 Low-Grade (Grade-2/3)	1 High-Grade (Grade 4 Astrocytoma)		The Area under the Curve (AUC)	Precision	Recall	F1 Score
Random Forest Classifier	10-fold cross validation	0.80	0.90	0.83 ± 0.16	0.81 ± 0.19	0.85 ± 0.13	0.93 ± 0.12	0.88 ± 0.11
Support vector Machine Classifier	10-fold cross validation	0.62	0.79	0.82 ± 0.14	0.82 ± 0.21	0.85 ± 0.13	0.91 ± 0.10	0.87 ± 0.09
Gradient boost Classifier	10-fold cross validation	0.96	0.98	0.71 ± 0.09	0.70 ± 0.17	0.80 ± 0.10	0.79 ± 0.13	0.78 ± 0.08
Naïve Bayes Classifier	10-fold cross validation	0.58	0.72	0.66 ± 0.18	0.71 ± 0.23	0.78 ± 0.06	0.72 ± 0.17	0.73 ± 0.14
Ada boost Classifier	10-fold cross validation	0.57	0.74	0.74 ± 0.19	0.75 ± 0.19	0.76 ± 0.09	0.79 ± 0.19	0.73 ± 0.13

Each performance value was calculated by averaging the results of the ten-fold cross-validation. HGG (grade 4 astrocytoma): class probability 1; low-grade glioma (grades 2/3 glioma): class probability 0.

## Data Availability

The data supporting the results are archived in the Tata Memorial Centre electronic data base. In order to maintain the privacy and confidentiality of patients, the data is anaonymised and only the Principal investigator and co-Principal investigators have access to the data. Anonymised data can be made available to individual researchers on request.
